# MicroRNA-Based Delivery Systems for Chronic Neuropathic Pain Treatment in Dorsal Root Ganglion

**DOI:** 10.3390/pharmaceutics17070930

**Published:** 2025-07-18

**Authors:** Stefan Jackson, Maria Rosa Gigliobianco, Cristina Casadidio, Piera Di Martino, Roberta Censi

**Affiliations:** 1 ChIP Chemistry Interdisciplinary Project Research Centre, School of Pharmacy, University of Camerino, Via Madonna delle Carceri, 62032 Camerino, MC, Italy; stefan.jackson@unicam.it (S.J.); roberta.censi@unicam.it (R.C.); 2 Department of Pharmacy, “G. D’Annunzio” of Chieti and Pescara University, Via dei Vestini 1, 66100 Chieti, CH, Italy; maria.gigliobianco@unich.it (M.R.G.); piera.dimartino@unich.it (P.D.M.)

**Keywords:** miRNA, dorsal root ganglia, drug delivery, gene delivery, biomaterials, polymers

## Abstract

Neuropathic pain is a significant global clinical issue that poses substantial challenges to both public health and the economy due to its complex underlying mechanisms. It has emerged as a serious health concern worldwide. Recent studies involving dorsal root ganglion (DRG) stimulation have provided strong evidence supporting its effectiveness in alleviating chronic pain and its potential for sustaining long-term pain relief. In addition to that, there has been ongoing research with clinical evidence relating to the role of small non-coding ribonucleic acids known as microRNAs in regulating gene expressions affecting pain signals. The signal pathway involves alterations in neuronal excitation, synaptic transmission, dysregulated signaling, and subsequent pro-inflammatory response activation and pain development. When microRNAs are dysregulated in the dorsal root ganglia neurons, they polarize macrophages from anti-inflammatory M2 to inflammatory M1 macrophages causing pain signal generation. By reversing this polarization, a therapeutic activity can be induced. However, the direct delivery of these nucleotides has been challenging due to limitations such as rapid clearance, degradation, and reduction in half-life. Therefore, safe and efficient carrier vehicles are fundamental for microRNA delivery. Here, we present a comprehensive analysis of miRNA-based nano-systems for chronic neuropathic pain, focusing on their impact in dorsal root ganglia. This review provides a critical evaluation of various delivery platforms, including viral, polymeric, lipid-based, and inorganic nanocarriers, emphasizing their therapeutic potential as well as their limitations in the treatment of chronic neuropathic pain. Innovative strategies such as hybrid nanocarriers and stimulus-responsive systems are also proposed to enhance the prospects for clinical translation. Serving as a roadmap for future research, this review aims to guide the development and optimization of miRNA-based therapies for effective and sustained neuropathic pain management.

## 1. Introduction

Pain is a widespread and burdensome health issue that significantly affects the global population. According to the International Association for the Study of Pain (IASP), pain is defined as “an unpleasant sensory and emotional experience associated with, or resembling that associated with, actual or potential tissue damage” [1]. It can be classified by duration into acute (short-term) and chronic (long-term) pain, and by cause into nociceptive (tissue damage) and neuropathic (nerve damage) pain. Pain signals originate from receptors in the peripheral neurons, which detect specific nociceptive inputs and transmit them to the spinal neurons, which then relay the signals to the brain for processing. Neuropathic pain occurs as a result of damage to the nerves. According to the IASP, it is defined as pain caused by a lesion or disease affecting the somatosensory nervous system. This type of pain affects approximately 7–10% of the global population and can significantly interfere with daily activities and the overall quality of life. Additionally, around 20–25% of the global population experiences chronic pain, and 20% of adults in Western countries report it as a major health concern [2,3,4,5,6]. Due to the multiple molecular mechanisms involved with neuropathic pain, there are many pre-existing clinical challenges. Furthermore, the currently available pain relief treatments such as surgery, physical therapies, psychological therapies, and medications, face serious challenges along with the extensive usage of medications like opioid or non-opioid drugs, resulting in side effects like drug addiction, abuse, and resistance or tolerance [7,8,9,10,11,12,13].

To overcome these issues, targeting the dorsal root ganglion has demonstrated potential effects on pain alleviation. The dorsal root ganglion (DRG) plays a vital role in neuropathic pain development, transmitting peripheral nociceptive input to the central nervous system. Studies, such as those by Berger et al., support DRG stimulation as a promising neuromodulation strategy for chronic pain management [14]. Pain was once thought to result solely from neuronal activation, but neurons are not the only contributors to pain signaling. In fact, glial cells also play critical roles: peripheral nerve injury activates spinal microglia and satellite glia in the DRG, which then release pro-inflammatory mediators that amplify pain signals [15].

Studies on small non-coding but functional RNAs called microRNAs (miRNAs), indicate miRNA upregulation or downregulation at the DRG following a nerve injury [16]. Molecular mechanisms underlying neuropathic pain involve miRNA dysregulation in DRG neurons, influencing neuronal excitability, synaptic transmission, and macrophage polarization from anti-inflammatory M2 to pro-inflammatory M1 phenotypes, thereby driving pain signaling [16,17,18,19]. At a molecular level, microRNAs act as key regulators of gene expression, orchestrating both immune and neuronal processes in the pain pathway [20]. A few of the miRNAs expressed in neuropathic pain conditions include miR-21, miR-30a, miR-124, miR-125, and miR-183 [21,22,23]. This can be manipulated and used from a therapeutic perspective. A few of the miRNA dysregulations utilized in association with neuropathic pain treatment at the DRG are listed in Table 1. Since miRNA recognition requires binding to a much shorter nucleotide seed sequence than an entire nucleotide as in the case of small interfering RNA (siRNA), the therapeutic index is larger comparatively [24,25].

Current treatments for neuropathic pain, such as opioid medications and surgical interventions, are often limited by issues like poor efficacy and the risk of addiction. In contrast, miRNA-based therapies show considerable potential by directly targeting the underlying molecular mechanisms of pain. However, successful clinical application remains challenging due to difficulties in delivering these therapies effectively, highlighting the need for advanced nanocarrier systems. This review presents a novel and comprehensive perspective by exploring two key areas. First, it investigates the pathophysiological functions of miRNAs in DRG-mediated pain. Second, it examines the development of innovative delivery systems designed to modulate miRNA activity within the DRG. The primary objective is to emphasize the central role of the DRG and miRNA in the onset and persistence of chronic neuropathic pain. Particular attention is given to the complex interactions among neuronal excitability, miRNA dysregulation, synaptic transmission, and inflammatory responses. In addition, the review discusses multiple therapeutic strategies that aim to modulate DRG function through targeted miRNA regulation. Furthermore, the limitations of delivering naked miRNA and an outline of how designing viral and non-viral delivery systems can overcome these barriers were also explored. By highlighting a comprehensive overview of polymeric, inorganic, lipidic, extracellular vesicle, and hydrogel-based delivery vehicles, a possible resolution to the pressing challenges in clinical translation, including immunogenicity, scalability, and long-term safety were addressed. Through these findings, we aim to provide an insight into how engineered miRNA-nano formulations pave the way for new avenues for safe and effective neuropathic pain therapies.

## 2. Role of the DRG in the Pain Pathway

**Figure 1:**
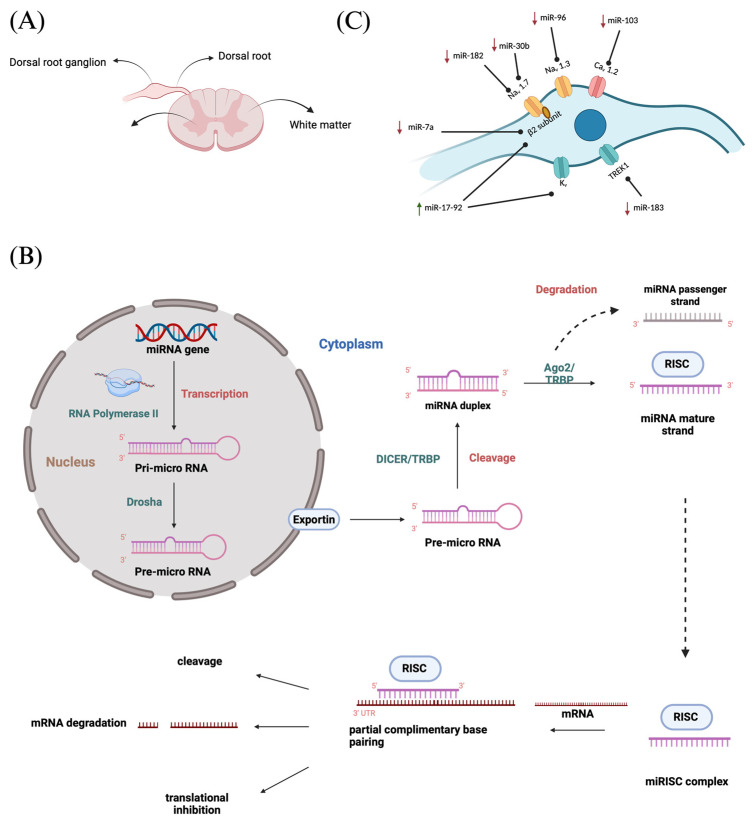
(**A**) Spinal cord cross section showing DRG; (**B**) MiRNA biogenesis—production of miRNAs and mechanism of gene expression regulation followed by miRNA–mRNA interaction resulting in translational repression or degradation of target mRNAs; (**C**) MiRNA regulation at DRG.

The DRG or spinal ganglion consists of a cluster of cell bodies, mainly of primary sensory neurons (PSNs) located within a sural sheath surrounded by cerebrospinal fluid (CSF) on the dorsal roots of spinal nerves, just outside the spinal cord (Figure 1A) and is involved in sensory signaling, modulation, and pain transmission [48,49]. Although these structures are approximately the size of a peanut, they contain around 15,000 neurons. They contain various other cell types such as satellite glial cells, endothelial cells, and macrophages along with PSNs [50]. In our human body, there are 31 pairs of spinal nerves of afferent sensory dorsal roots and motor ventral roots which make up the spinal cord. Between adjacent vertebral segments, there are intervertebral foramina from which these spinal nerves emerge. A dorsal root that exits from the intervertebral foramina, when merging with a ventral nerve root, forms the DRG [14]. It accommodates cell bodies of PSNs such “A-neurons” or large-light neurons which transmit non-noxious signals and “C-neurons” or small-dark neurons that transmit noxious signals [51]. Each cell body in the dorsal root ganglia is a modified bipolar neuron known as a pseudo-unipolar neuron. It has a single axonal process that bifurcates into a T-shaped structure, consisting of a central branch that enters the spinal cord and a peripheral branch that connects to somatic and visceral sensory receptors. These pseudo unipolar neurons are associated with the development and maintenance of neuropathic pain since the glial cells in DRGs respond to a nerve injury and produce inflammatory markers [14].

## 3. MicroRNA Biogenesis and Its Role in the Pain Signal Pathway

### 3.1. MicroRNA Biogenesis

The miRNAs are a class of small non-coding RNA (ncRNA) having 21 to 25 nucleotides in length. They are partially complementary to one or more messenger RNA (mRNA) molecules and have a pivotal function in post transcriptional gene regulation, translational repression, mRNA cleavage, and deadenylation [51,52,53,54]. The biogenesis of miRNA involves a series of steps such as the transcription of miRNA genes to primary miRNA precursors called pre-miRNAs by RNA polymerase II, which further becomes cleaved by Drosha to form 70-nucleotide hairpin structures known as pre-miRNAs. Then, exportin-5 exports the pre-miRNA out of the nucleus to the cytoplasm where dicer and trans-activation response RNA-binding protein (TRBP) converts it to a 20- to 24-nucleotide double-stranded duplex of mature miRNA strand and a complementary or designated miRNA strand (miRNA-miRNA*) [55,56,57,58]. Subsequently, the mature strand (guide strand) is incorporated into the RNA-induced silencing complex (RISC) by Ago2 and TRBP to form miRISC, and the passenger strand (designated strand) becomes degraded (Figure 1B) [59]. Finally, miRISC binds with target mRNA to induce either protein synthesis interference or degradation of the target mRNA [60]. They act as gene-regulating modulators by post-transcriptionally inhibiting protein expression in targeted mRNAs via 3′-UTR recognition of mRNA in a sequence specific approach [61].

### 3.2. MicroRNA Regulation of the Pain Signaling Pathway

Understanding the involvement of miRNA in pain pathways enables the identification of novel targets for neuropathic pain. Dynamic gene-regulator-altered miRNA profiles are often implicated in neuropathic pain pathogenesis. For instance, a recent transcriptomic study by Xiong et al. analyzed dorsal root ganglia after chronic constriction injury and found 47 differentially expressed miRNAs; enriched pathways included Rap1, Ras, and Hippo signaling, suggesting broad miRNA-mediated changes in neuropathic pain [62]. These non-coding RNAs that fine-tune gene expression, and alter miRNA profiles are often implicated in the pathogenesis of neuropathic pain. From neuronal excitability and ion channel expression to neuroinflammation and synaptic plasticity, these molecules regulate diverse processes and together shape pain signaling [63]. Following peripheral nerve injury, aberrant miRNA activity can disrupt normal pain transmission, leading to the hyperexcitability of neurons and amplification of pain signals. Based on the signaling pathway, the role of miRNAs can be categorized accordingly.

#### 3.2.1. miRNA Regulation of Ion Channels and Neuronal Excitability

Various ion channels present along the membrane allow the propagation of action potentials via the axon of sensory neurons and transmit electrical impulses from the body to the brain. Following a nerve injury, miRNAs alter the expression in these ion channels, thereby resulting in neuronal excitability and amplified pain perception. Zhao et al. observed that miRNAs play pivotal roles in the development and progression of neuropathic pain by influencing neuronal excitability and plasticity, modulating ion channels, regulating neuroinflammatory responses, and affecting synaptic plasticity, among other critical functions [63]. Ion channels such as voltage-gated sodium channels (VGSCs) and their nine subtypes (Na_v_ 1.1–Na_v_ 1.9), along with calcium channels (Ca_v_), potassium channels (K_v_) as well as TREK1 (a mechano-temperature-sensitive potassium channel), play a vital role in neuronal excitability because of miRNA regulation (Figure 1C) [64]. VGSCs critical in sensory neuron firing, are particularly influenced by miRNAs. Golmakani et al. (2024) highlighted that increasing miR-30b (which targets the Na_v_ 1.7 sodium channel) or miR-7a (targeting sodium channel β2 subunits) inhibits pain [65]. MiR-30b overexpression was found to directly downregulate Na_v_ channel expression (including Na_v_ 1.3, Na_v_ 1.6, and Na_v_ 1.7) in injured neurons, thereby reducing neuropathic pain behaviors [30,66,67]. Likewise, miR-7a represses the β2 subunit of VGSCs (an auxiliary subunit encoded by SCN2B), normalizing the hyperexcitability of nociceptive neurons after nerve injury [26]. Consistent with these findings, other miRNAs (miR-96 and miR-182) also suppress Na_v_ channel isoforms to curb repetitive firing [32,41,68]. In addition, a cluster of miRNAs known as miR-17-92 are downregulated in certain neuropathic pain models; when active, this cluster can reduce potassium channel (K_v_) currents and thus increase neuronal firing, whereas blocking miR-17-92 alleviates allodynia by restoring the K^+^ channel function [27]. Similarly, miR-137 inhibition has been shown to normalize K_v_1.2 channel levels and DRG neuron firing, reversing pain phenotypes [34,69]. Changes in other miRNA families (for example, the let-7 family) have also been linked to the altered expression of ion channels and heightened sensory neuron activity, further underscoring that dysregulated ion channel expression due to miRNA imbalances is a key mechanism of neuropathic pain.

#### 3.2.2. miRNAs in Neuroinflammation, Glial Signaling, and Microglial Activation

Neuroinflammation is a driving force in chronic pain, and miRNAs are pivotal regulators of the inflammatory pathways in the nervous system. Following nerve injury, activated immune cells (microglia, macrophages) and astrocytes release pro-inflammatory cytokines that sensitize neurons. Certain miRNAs act as breaks on this process by targeting inflammatory signaling molecules. For instance, miR-146a-5p is known to target IRAK1 and TRAF6, adaptor proteins in the Toll-like receptor (TLR) and IL-1 receptor pathways. The upregulation of miR-146a-5p has been shown to dampen these pathways: in a rat chronic constriction injury (CCI) model, the intrathecal delivery of a miR-146a-5p agomir (mimic) significantly alleviated mechanical allodynia and thermal hyperalgesia by suppressing the injury-induced increase of IRAK1 and TRAF6 in the DRG and spinal cord [39]. Conversely, blocking miR-146a worsened pain and elevated IRAK1/TRAF6 levels, confirming that miR-146a-5p’s anti-inflammatory action can prevent neuropathic pain development. In cultured microglial cells, miR-146a overexpression reverses pro-inflammatory markers (Arg-1, IL-10) [70]. Similarly, miR-216a-5p promotes an anti-inflammatory phenotype in microglia via the TLR4 pathway. Upregulating miR-216a-5p was found to inhibit the HMGB1–TLR4–NF-κB signaling cascade, leading to the reduced release of IL-1β, IL-6, and TNF-α and a shift of microglia from the pro-inflammatory M1 state to anti-inflammatory M2 [43,71]. This change corresponds with the prevention of neuropathic pain in spinal cord injury models, positioning miR-216a-5p as a potential therapeutic target for regulating neuroinflammation.

Activated microglia and macrophages themselves are modulated by miRNAs, creating a feedback loop in pain regulation. One notable regulator is miR-124, a microRNA highly enriched in resting (M2-like) microglia. MiR-124 helps keep microglia in an anti-inflammatory state by inhibiting targets such as GRK2 (a kinase that drives pro-inflammatory signaling in glia). Loss of miR-124 after nerve injury has been associated with a skewing of microglia toward the M1 state, exacerbating neuroinflammation and pain. In line with this, restoring miR-124 levels produces analgesic effects: intrathecal administration of miR-124 (or mimetics) reduced neuropathic pain and inflammation by shifting microglial polarization toward the M2 state [33]. For example, here, Willemen et al. found that chronic pain models show reduced miR-124 in spinal microglia and a predominance of M1 markers, whereas intrathecal miR-124 fully prevented the transition to persistent pain and normalized the spinal M1/M2 marker balance. MiR-124 directly suppresses GRK2 expression and thereby modulates the microglial M1/M2 balance. The importance of such non-neuronal regulation is further illustrated by miR-124’s absence leading to unchecked M1 activation and sustained pain. Additionally, miR-146a, miR-216a, and miR-124 are often co-regulated in neuropathic conditions, and their collective downregulation in injured DRGs and spinal cord correlates with heightened cytokine levels and persistent pain [63,72]. Therapeutically boosting these anti-inflammatory miRNAs could thus attenuate the neuroimmune activation underlying chronic pain.

Not only do miRNAs within immune cells matter, but neurons and glia also communicate via miRNA-containing extracellular vesicles, amplifying neuroinflammatory signals. DRG neurons can secrete exosomes or microvesicles that carry specific miRNAs to neighboring immune cells. A striking example is miR-23a: after nerve injury, DRG sensory neurons markedly upregulate miR-23a and package it into exosomes. These miR-23a-rich vesicles are released into the extracellular space and subsequently taken up by macrophages at the injury site [29]. Once inside macrophages, miR-23a binds to and inhibits *A20* (TNFAIP3), a negative regulator of NF-κB signaling. The result is enhanced NF-κB activity and a shift of macrophages toward the pro-inflammatory M1 phenotype (with increased Nos2/iNOS and reduced Mrc1/CD206 expression). This neuron-to-immune transfer of miR-23a thereby promotes the release of cytokines that drive neuropathic pain. Importantly, disrupting this pathway has analgesic effects: delivering an exosomal miR-23a antagomir (an inhibitor) in vivo blunted NF-κB activation, reduced M1 macrophage accumulation in the DRG, and attenuated neuropathic hypersensitivity [29]. These findings reveal a novel non-neuronal communication route in pain: injured neurons actively influence immune cells via miRNAs, adding another layer of complexity to pain signaling. Notably, directly inhibiting pro-nociceptive miRNAs (anti-miR therapy) has shown benefits: for example, intrathecal miR-21 antagomir reduced hyperalgesia and shifted macrophages towards an anti-inflammatory phenotype [28,73]. Overall, by controlling cytokine networks, glial activation states (including microglial M1/M2 polarization), and neuron–immune crosstalk, miRNAs serve as critical modulators of neuroinflammation in chronic pain states.

#### 3.2.3. miRNAs in Neuronal Survival and Regeneration

Beyond excitability and inflammation, miRNAs also influence neuron-intrinsic processes like survival, axon regeneration, and synaptic remodeling that can affect chronic pain development. Nerve injury triggers regenerative programs in neurons as well as stress responses (e.g., apoptosis), and miRNAs help govern these outcomes. One well-studied example is miR-21, which is rapidly upregulated in injured DRG neurons and in the surrounding glial cells. MiR-21 is known to promote pro-regenerative signaling in part by targeting the PTEN/Akt/mTOR pathway, a key pathway that, when disinhibited, allows neurons to grow axons and resist apoptosis [74]. Other miRNAs predominantly support the regenerative and survival side. For instance, miR-125b is upregulated in models of nerve injury and has been shown to enhance axon regeneration after spinal cord trauma by suppressing inhibitors of the JAK/STAT3 pathway [75]. The overexpression of miR-125b reduces neuronal apoptosis and inflammation, thereby creating a neuronal protective effect. Similarly, miR-210 is induced as a negative feedback regulator in injured tissues; it targets components of the NF-κB pathway and HIF-1α, thereby reducing inflammatory damage and supporting cell survival [76]. In a spinal injury model, treatment with agomiR-210 decreased levels of IL-1β and TNF-α and protected neurons, indicating its role in counteracting inflammation-related neurotoxicity [77]. Another example is miR-181c-5p, which has been shown to inhibit the pro-apoptotic factor BIM; the upregulation of miR-181c-5p effectively reduced neuron apoptosis and promoted axon regrowth in injured neural tissue [78]. Likewise, miR-138 has been implicated in regenerative mechanisms: an early study found that miR-138 forms a regulatory loop with SIRT1, a deacetylase that promotes axon outgrowth, suggesting that downregulating miR-138 may release pro-regenerative factors and enhance neurite extension [79]. In summary, numerous miRNAs become dysregulated after nerve injury not only in ways that exacerbate pain, but also as part of the attempt to recover neural function.

## 4. MiRNA Delivery Systems

From recent studies, it has been estimated that miRNAs regulate more than 30% of the protein-coding genes associated with various biological processes like cell proliferation, apoptosis, fat metabolism, cell differentiation, and disease progression [80,81]. The recent surge of interest in therapies based on these non-coding RNAs over the past few decades is because of their direct or indirect involvement in various biological anomalies [82,83]. MiRNA mimics or antagomirs (anti-miRNAs) are used as therapeutic agents either to restore or suppress the gene expression [84,85,86,87]. Despite all these factors, there are major limitations or challenges corresponding to the delivery of miRNA. One of the major drawbacks is the inherent instability of miRNA in biological fluids, in which it is susceptible to rapid degradation by nucleases [88]. This degradation in turn results in a significant reduction in the half-life of free miRNAs, thus reducing their therapeutic potential. For an effective therapeutic action, miRNA has to be delivered into the cytoplasm of the target cells in order to interact with the RISC. Navigating through complex biological barriers such as cell membrane and the endosomal compartment is very difficult for a naked miRNA. Therefore, free miRNAs often end up entrapped in endosomes and results in subsequent degradation [89]. Apart from that, rapid clearance from blood, low permeability, and other extracellular barriers such as the reticuloendothelial system (RES) can shorten the half-life of a short ncRNA (Table 2). The negative charge of miRNA poses significant challenges especially in terms of electrostatic repulsion when it comes to cell membrane which is negatively charged due to the presence of phospholipids [90]. This negative charge not only hinders cell membrane penetration but hinders its ability to be internalized by cells and thus reduces the efficiency of delivery. It may also lead to unwanted interactions with positively charged serum proteins leading to miRNA sequestration and reduction in bioavailability as well as therapeutic efficacy. Another challenge is in the form of cations present in biological fluids that may potentially lead to the aggregation of these negatively charged miRNA molecules and reduction in the functional concentration of miRNA, thus complicating its delivery [91]. The ability of miRNAs to bind to multiple mRNA targets may result in unintended interactions or off-target effects and disrupt the expression of genes. Chemically modified or vectorized miRNAs on the other hand face intracellular barriers and intracellular trafficking along with potential issues of diffusion through membranes because of the high molecular weight of the modified miRNAs. Therefore, miRNA needs to be encapsulated and delivered using an optimized delivery vehicle to surpass all the above-mentioned limiting factors.

**Figure 2:**
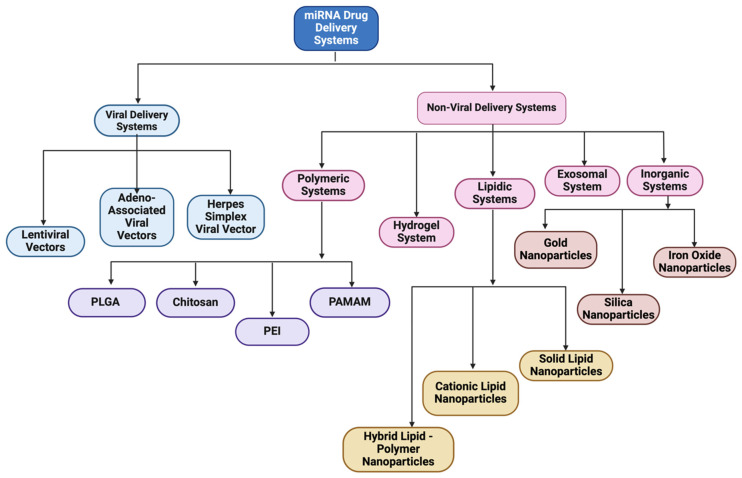
Classification of miRNA-based drug delivery systems.

To combat all the limitations associated with the naked delivery of miRNA, a carrier is required which ideally must be biocompatible, biodegradable, non-toxic, non-immunogenic but at the same time protect the genetic material from enzymatic degradation or clearance and display a positive surface charge, along with an optimum size ranging between 50 and 200 nm (Table 2). The positive surface charge helps to overcome the electrostatic repulsion between the negatively charged miRNA and the cell membrane and increase the binding efficiency, thereby protecting it from degradation by nucleases, hindering interactions with serum proteins and allowing endosomal escape via the proton sponge effect [24]. The particle size is often a factor that determines the circulation time, tissue distribution, cellular uptake, and the ability to cross biological barriers. For efficient cellular uptake, particles below 200 nm are effectively internalized by cells via endocytosis, while larger particles often tend to be cleared more quickly by the body [92]. If particles are too small, i.e., less than 10 nm, they may not be able to carry enough cargo or may possibly be excreted too quickly through renal clearance [93]. Along with that, this smaller particle size offers prolonged circulation and better tissue penetration and minimizes aggregation. Furthermore, miRNA must be internalized by the cells and released into the cytoplasm. This is where a carrier system comes into play. There are mainly two types of delivery systems: viral and non-viral carriers (Figure 2). Viral-based systems include the use of retroviruses, lentiviruses, and adenoviruses [94]. A few among the non-viral vectorization materials that can be used as a delivery system includes polymeric, inorganic, lipidic, and extracellular vesicle-based carrier systems [95]. A summary of current miRNA-based delivery systems targeting the DRG for chronic neuropathic pain management is presented in Table 3.

### 4.1. Viral Delivery Systems

Lentivirus, adeno-associated virus, and herpes simplex virus are the most common viral vectors having high transfection efficiency along with a high level of constant expression of miRNA mimics or antagomirs that have modifications in certain specific genomic areas, in order to prevent replication [103].

#### 4.1.1. Lentiviral Vectors

**Figure 3:**
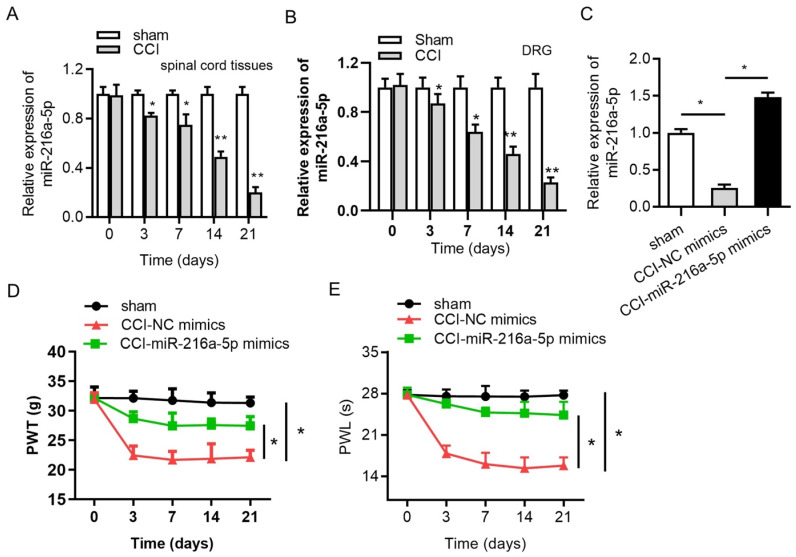
RT-qPCR analysis of expression of miR-216a-5p in (**A**) spinal cord, (**B**) DRG in sham, and CCI rat models (**C**). Overexpression efficiency (**D**) effect of overexpression of miRNA on mechanical allodynia. (**E**) Effect of overexpression of miRNA on thermal hyperalgesia; * *p* < 0.05, ** *p* < 0.01 (reprinted from [43] with permission from Elsevier, Amsterdam, The Netherlands).

Lentivirus is a subgroup of retroviruses that can transfect both dividing and non-dividing cells and have the ability to transfer and integrate about 8 kb of foreign genetic sequences into the host genome [104]. This viral system offers high transfection efficiency and the long-term stable expression of the incorporated miRNA making them a potential candidate for treating neurologic disorders. A study based on the functional recovery of injured spinal cord in mice using lentivirus-mediated delivery of miR-133b confirmed the role of miRNA in neurite growth enhancement, proving its high therapeutic potential [105]. Lentiviral vector-based delivery of miR-145 in another study demonstrated regulation of human corneal epithelial differentiation [106]. Li H et al. based on a CCI (chronic constriction injury)-induced neuropathic pain model studied the regulatory mechanism of jagged 1 (JAG1) by miR-124-3p and miR-141-3p in neuropathic pain in the spinal cord dorsal horn of rats using recombinant lentivirus [107]. Lentivirus-mediated gene transfer involving the overexpression of miR-142-3p and miR-141 in SNL (spinal nerve ligation) and CCI models, respectively, demonstrated alleviation of neuropathic pain via inhibiting high-mobility group box 1 (HMGB1) [36,37]. Another study based on the upregulation of miR-144 using lentiviral vector in a CCI-induced model confirmed its therapeutic potential via the evaluation of the paw withdrawal threshold (PWT) and paw withdrawal latency (PWL) for neuropathic pain treatment [38]. An experimental study by Shi et al. demonstrated that delivering miR-145 mimics to dorsal root ganglion (DRG) neurons via lentiviral vectors directly targeted Akt3 and inhibited downstream signaling pathways involving nuclear factor kappa B (NF-κB) and the mammalian target of rapamycin (mTOR), resulting in reduced mechanical allodynia and thermal hyperalgesia [96]. Inhibition of X-inactive specific transcript (XIST) by the downregulation of ZEB1 and upregulation miR-150 regulation was studied by Yan et al. on CCI-induced rat models as an axis to provide a therapeutic target for neuropathic pain [97]. In another lentiviral-based delivery of miRNA by Shi et al., alleviation of neuropathic pain was studied on CCI rat models via the inhibition of the TWIK-related potassium channel 1 (TREK-1) channel using miR-183-5p [42]. Another experimental work based on the overexpression of miR-216a-5p using lentiviral vector on CCI rat models attenuated neuropathic pain and improved mechanical allodynia and thermal hyperalgesia [43]. Here, miR-216-5p alleviates neuropathic pain by targeting lysine demethylase 3A (KDM3A) and by inactivating the Wnt/B-catenin signaling pathway (Figure 3).

#### 4.1.2. Adeno-Associated Viral Vectors

Adeno-associated viruses (AAVs) are safe adenoviral vectors due to their deficiency in replication and pathogenicity [108]. Compared to adenoviruses that are double-stranded DNA viruses containing more than 100 serotypes, AAVs are single-stranded DNA parvoviruses from the genus *Dependovirus*, which consists of 12 primate serotypes (AAV1 to AAV12) [109]. AAVs can cause long-term expression in vivo due to their capability of integrating into a specific site on chromosome 19 without any noticeable effects [94]. The disadvantage of AAVs is associated with their limited transgene capacity of 4.8 kb that limits its applications for large gene delivery, but miRNA, due to its small size, often uses AAVs as a delivery system. They have the ability to transfect both dividing and non-dividing cells.

**Figure 4:**
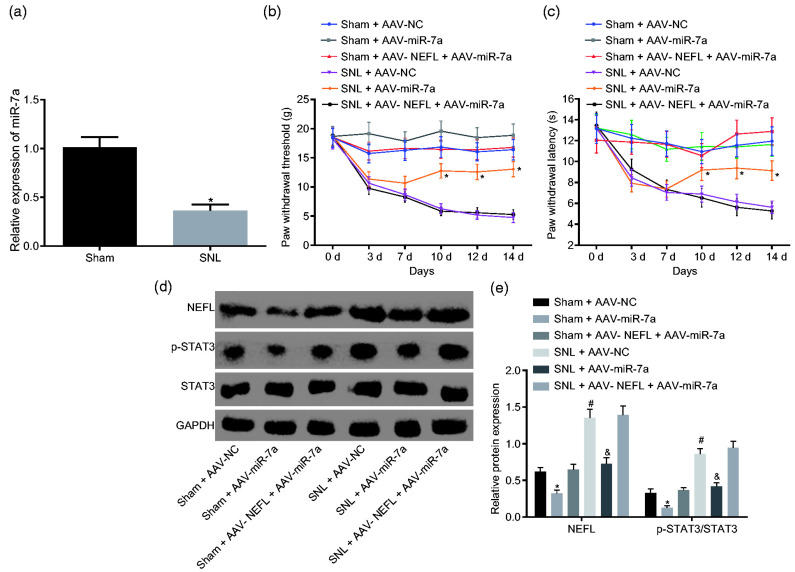
Overexpression of miR-7a attenuates neuropathic pain by suppressing NEFL expression and the STAT3 signaling pathway. (**a**) miR-7a expression in sham and SNL-treated rats; * *p* < 0.05 versus the sham group;. (**b**) PWT of sham/SNL models post AAV-miR-7a infection after operation. (**c**) PWT of sham/SNL models in response to infection of AAV-miR-7a after operation (**d**,**e**) protein bands and levels of NEFL, STAT3, and extension of STAT3 phosphorylation in SNL-treated rats in response to infection of AAV-miR-7a at the 14th day after operation; * *p* < 0.05 versus the sham + AAV-NC group; ^#^ *p* < 0.05 versus the SNL + AAV-NC group; ^&^ *p* < 0.05 versus the sham + AAV-NEFL + AAV-miR-7a group (reprinted from [98]).

An investigation on the effects of miR-7a in a spinal SNL rat model using an AAV inhibited the activation of the signal transducer and activator of the transcription 3 (STAT3) signaling pathway and relieved neuropathic pain, proving it to be a significant and potential treatment for neuropathic pain patients (Figure 4) [98]. Another study on miR-7a by Sakai A et al. in an SNL model using male Sprague-Dawley rats demonstrated the alleviation of neuropathic pain via the regulation of neuronal excitation [26]. MiR-17-92, an miRNA cluster based on other experimental research, was found to regulate the voltage-gated potassium channels associated with chronic neuropathic pain [27]. Similarly, Jung et al. delivered an AAV-encoding shRNA targeting GCH1, a pain-related enzyme, to the DRG. They observed that AAV-shGCH1 deactivated microglia and protected against the development of neuropathic pain [110].

#### 4.1.3. Herpes Simplex Viral Vectors

Herpes simplex virus (HSV) is a large DNA virus with the capacity to carry multiple therapeutic genes. Its natural ability to infect axonal nerve terminals at peripheral sites makes it an ideal vector for gene delivery to the nervous system. Unlike lentivirus, adeno-associated virus, and retrovirus that only have a small genome-packing capacity of less than 8 kb, HSV can accommodate up to a 150 kb genome [111]. Studies conducted by Chattopadhyay et al. demonstrated that HSV vector-mediated delivery of miRNA into DRG neurons in vivo inhibits voltage-gated sodium channels thereby reducing painful diabetic neuropathy [89]. In this study, streptozotocin (STZ)-induced painful diabetic neuropathy (PDN) male Sprague-Dawley rat models were inoculated with the vector containing the miRNA sequence (QHmiNa_v_) 2 weeks after the onset of diabetes and it resulted in a reduction in Na_v_α subunit levels in DRG neurons as well subsequent reduction in cold allodynia, thermal hyperalgesia, and mechanical hyperalgesia.

### 4.2. Non-Viral Delivery Systems

Even though viral delivery systems offer high transfection efficiency, often, applications are limited due to potential issues associated with immunogenic and inflammatory responses, low loading capacity, and quality control [112]. On the other hand, non-viral delivery systems offer lower toxicity and reduced immunogenicity, and they are not restricted by transgene size limitations inherent to viral delivery systems [94]. Some of the common non-viral carriers include polymeric, inorganic, lipidic, extracellular vesicle, or hydrogel-based systems.

#### 4.2.1. Polymeric Nano-Systems

Polymeric nanoparticles can be obtained from either naturally occurring polymers such as chitosan and dextran or from synthetic polymers, namely poly(lactic-co-glycolic acid) (PLGA), polyamidoamine (PAMAM), polyethyleneimine (PEI), polylysine (PLL), poly[2-(dimethylamino) ethyl methacrylate] (pDMAEMA), etc. Polymeric nanoparticles are among the most promising nano-delivery systems, owing to their simple synthetic procedures, structure, transfection efficiency, non-immunogenicity, and biocompatible nature [113]. The most common forms include nanospheres and nanocapsules having subclasses such as polyplexes, polymerosomes, and dendrimers.

Polyplexes comprise cationic polymers that can bind with nucleic acids to form compact structures via electrostatic interactions. It is a spontaneous process in which typically, an excess of cationic polymer is used compared to that of the nucleotide (amine to phosphate (N/P) ratio > 1) in order to condense the nucleic acid cargo and have nanoparticles with a positive surface charge and small particle size [114,115]. The negatively charged nucleic acids are well protected within the polymer matrix by the polymer chains from nucleolytic enzymes, and the stability of the formed nanoparticles can be enhanced by the incorporation of covalent cross-linkers or by the addition of an alkyl group [116,117]. The formation of polymerosomes or polymer vesicles on the other hand involves the self-assembly of amphiphilic block co-polymers via the hydrophobic effect. Some of the methods are the direct dissolution of a copolymer, film rehydration, solvent displacement, and probe sonication [118]. Based on the hydrophilic nature which helps in the self-assembling of the block copolymers to form polymerosomes, nucleic acids are well encapsulated in the inner aqueous core of the vesicles. On the other hand, dendrimers can be synthesized by an iterative sequence of growth and activation steps either by divergent or convergent growth approach methods [119]. Every successive reaction leads to the generation of a new branch and thus repeating the number of cycles to form a dendrimer generation. By tuning the dendrimer generation, the mass and the size of the dendrimers can be manipulated, and its surface can be functionalized by the conjugation of different moieties to its reactive terminal groups, causing them to be good gene delivery platforms. The conjugation of hydrophilic polymer chains such as polyethylene glycol (PEG) can improve the stability and biocompatibility of the polyplex and dendrimer systems, by reducing the particle aggregation and preventing coalescence [120,121].

##### PLGA-Based Nano-System

PLGA is a copolymer produced as a result of catalyzed ring-opening copolymerization of poly-lactic acid (PLA) and poly-glycolic acid (PGA) which decomposes to non-toxic products such as carbon dioxide and water via the Krebs cycle and is eliminated from the body [122]. PLGA has potential drug delivery applications since it is part of formulations that obtained US Food and Drug Administration (FDA) and European Medicine Agency (EMA) approval [123]. The individual monomer unit, PLA, is a hydrophobic polymer with low mechanical strength. On the other hand, PGA is a crystalline and hydrophilic moiety with a fast degradation rate and low water solubility. The properties of PLGA co-polymer depends on the ratio LA/GA of the individual monomer units and the molecular weight of PLGA [124]. An increase in the ratio results in the escalation of overall hydrophobicity, leading to a low degradation rate and slower drug release rate. On the other hand, the lowering of the molecular weight increases the degradation and drug release rates. It has been observed from studies that an increase in the molecular weight also results in nanoparticles with a larger size [125,126]. These parameters based on hydrophobicity, release profile rates, as well as drug loading efficiency, are crucial for formulating a PLGA nanoparticle.

**Figure 5:**
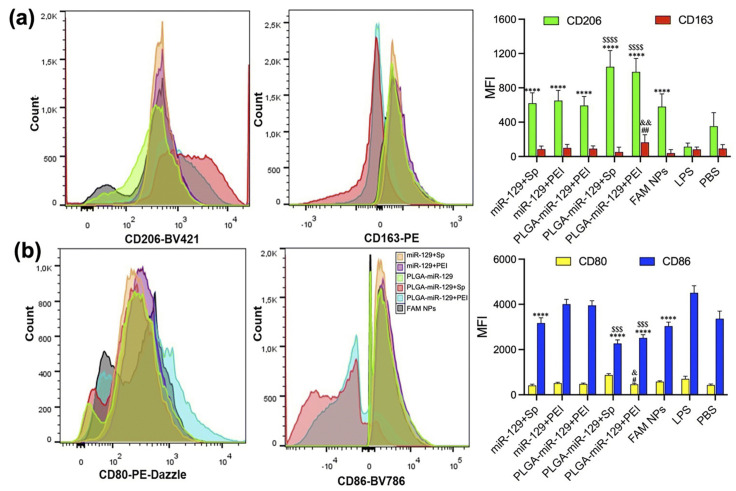
Flow cytometry analysis, indicating the modulation of activated BV-2 cells. (**a**) Significant upregulation of pro-regenerative markers CD206 and CD163 in all nanoformulations compared to LPS group. **** *p* < 0.0001 vs. LPS group and ^$$$$^ *p* < 0.0001 vs. miR-129+Sp, miR-129+PEI, PLGA-miR-129, and FAM NPs. CD163; ^##^ *p* < 0.01 vs. LPS group and ^&&^ *p* < 0.01 vs. miR-129+Sp, miR-129+PEI, PLGA-miR-129, PLGA-miR-129+Sp, and FAM NPs. (**b**) Downregulation of pro-inflammatory markers CD80 and CD86 in all nanoformulations. ^#^ *p* < 0.05 vs. LPS group and ^&^ *p* < 0.05 vs. miR-129+Sp, miR-129+PEI, PLGA-miR-129, PLGA-miR-129+Sp, and FAM NPs. CD86; **** *p* < 0.0001 in response to miR-129+Sp, PLGA-miR-129+Sp, PLGA-miR-129+PEI, and FAM NPs compared to LPS group and ^$$$^ *p* < 0.001 vs. miR-129+Sp, miR-129+PEI, and PLGA-miR-129 (reprinted from [130]).

Even though PLGA is biodegradable and non-toxic for a wide variety of clinical applications, its hydrophobic properties along with the subsequent issues of opsonization associated with its intravenous administration make it unsuitable for the effective delivery of miRNA. To prevent this, modifications such as a coating with PEG, which is biocompatible, hydrophilic, non-toxic, and FDA approved, helps to shield the carriers and to enhance permeability and retention [24]. Two main synthesis procedures for PLGA-PEG nano-systems include nanoprecipitation and a double emulsion solvent evaporation method. In the nanoprecipitation method, PLGA and PEG are dissolved in a suitable solvent and added to an aqueous phase for self-assembly, followed by solvent removal via dialysis or volatilization and finally nanoparticle collection. On the other hand, the double emulsion solvent evaporation method involves the addition of PLGA, PEG, and drug in an organic solvent to prepare water in an oil emulsion. The addition of this resultant emulsion into a water phase followed by homogenization and sonication results in a water in oil in water (W/O/W) emulsion [127]. Organic solvent evaporation and subsequent filtration yield drug-loaded PLGA-PEG nanoparticles. Compared to that of a single emulsion solvent evaporation method, this improves the entrapment efficiency as well as sustained release properties of the nano-system [128]. Pham et al., based on a study on miRNA 146a-5p-loaded PLGA nanoparticles in a rat spinal nerve ligation-induced neuropathic pain model, concluded that PLGA nanoparticles were a promising drug delivery platform by improving pain behaviors through the inhibition of multiple inflammatory pathways and the subsequent reduction in proinflammatory cytokine release [129]. In the study, PLGA nanoparticles were prepared via double emulsion through sonication of the primary emulsion containing the miRNA drug in TE7.5 buffer and PLGA dissolved in dichloromethane (DCM), with a secondary water phase containing poly vinyl alcohol (PVA) to form W/O/W. A study based on miRNA-129-5p encapsulated in PLGA nanoparticles by Kalashnikova et al. proved the role of miRNA in the regulation of microglial activation and modulation of innate immune cells as well as inhibited neuroinflammation after CNS injury [130]. Studies were conducted on BV-2 cells, which showed the substantial upregulation of pro-regenerative markers and downregulation of pro-inflammatory markers (Figure 5).

##### Chitosan-Based Nano-System

Chitosan, a derivative of N-deacetylation of chitin, a naturally occurring polymer, is a polysaccharide co-polymer having monomer units, D-glucosamine and N-acetyl-D-glucosamine [131]. It is biocompatible, biodegradable, and has FDA GRAS (generally recognized as safe) status. Recent in vitro and in vivo reports suggest that chitosan-based nanocarriers elevate nitric oxide production and thereby showcase wound healing properties as well [132,133]. A few chitosan-based nanocarriers are undergoing pre-clinical and early clinical development with a minimum of 15 clinical trials, making chitosan-based nano-systems an intervention that can provide safe drug delivery in the near future [134].

Various parameters affect the chitosan-miRNA nanoparticles such as molar mass, degree of deacetylation, and pH. A short-chain-length chitosan with less than a 15 kDa molar mass cannot condense the genetic material and complete the binding to form an intact nanoparticle [95]. On the other hand, chitosan with a molar mass greater than 300 kDa may have a stronger binding affinity with the genetic material that could potentially challenge the gene release [135]. A molar mass 5 to 10 times higher than the nucleotide RNA are required to form compactly structured nanoparticles of a suitable size via electrostatic forces [136]. The commonly used molar mass of chitosan is around 100 kDa, which can formulate nanoparticles of a size less than 200 nm which are safely tolerated by cells in order to have a potential biological effect.

**Figure 6:**
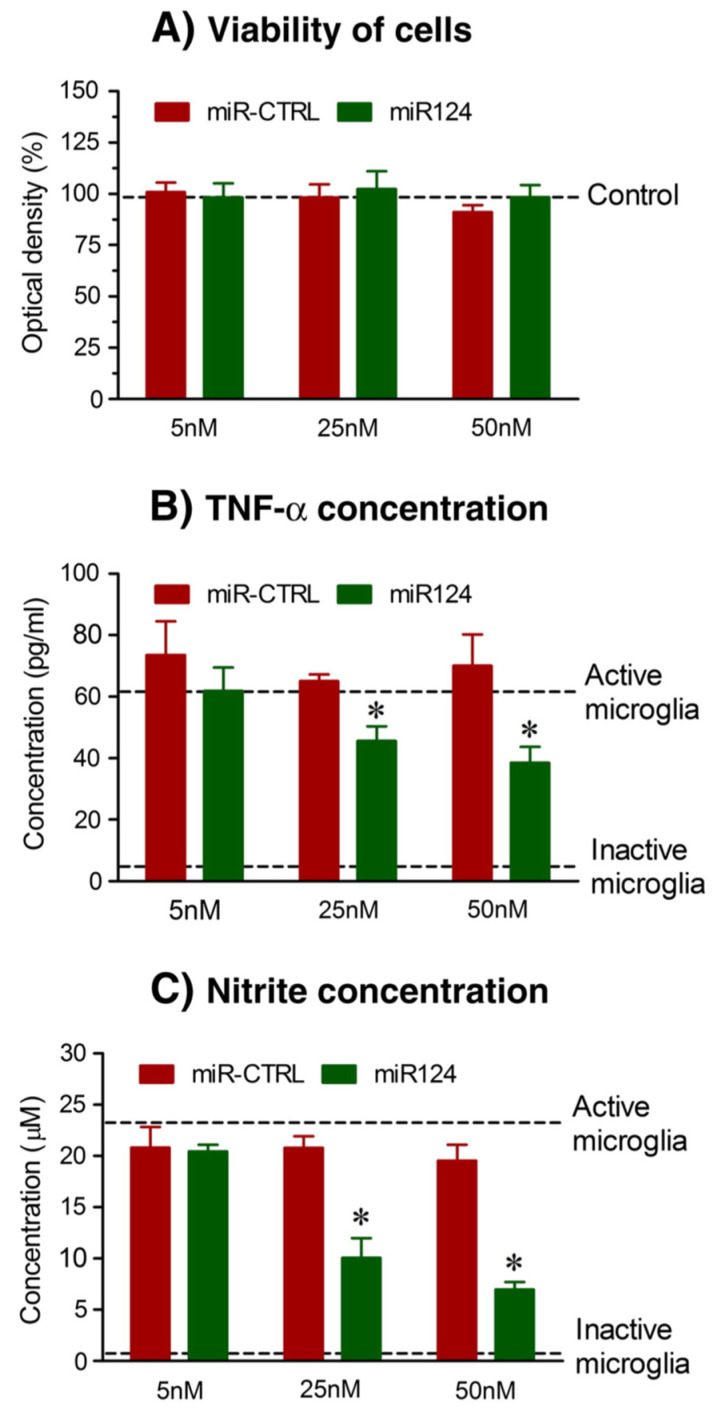
(**A**) Cytotoxicity of chitosan/miRNA particles measured by AlamarBlue^®^ viability assay indicate no significant decrease in viability after treatment with chitosan particles. MiR124-loaded chitosan polyplex particles reduce inflammatory factors like (**B**) TNF-α and (**C**) nitrite species; * *p* < 0.01 and 0.05 versus LPS and IFN-γ alone (reprinted from [140] with permission from Elsevier).

The main chitosan–miRNA-based nanocarrier formulation methodologies involve simple complexation and an ionic gelation method. Mixing of miRNA and chitosan at an appropriate N/P ratio and weight ratios to obtain biologically active nanoparticles forms the basis of simple complexation [137]. On the other hand, ionic gelation is based on the electrostatic interaction between the anionic and cationic charges of miRNA and chitosan, respectively, in the presence of a cross-linking agent such as tripolyphosphate (TPP), which helps in strengthening and stabilizing the chitosan–miRNA interactions [138]. The conjugation of chitosan to tragacanthic acid (TA) and glutathione was used in a study by Shamaeizadeh et al. to deliver miR-219a-5P to treat multiple sclerosis, a chronic neurodegenerative disease [139]. Louw A et al., in a study based on chitosan polyplex-mediated delivery of miR-124, demonstrated an alteration in the inflammatory response in spinal cord injury models (Figure 6) [140].

##### PEI-Based Nano-Systems

PEI is a cationic synthetic polymer rich in amine groups, that can condense nucleic acids to form nanoparticles via electrostatic interaction, thereby protecting nucleic acid from degradation and facilitating the cellular uptake of these nucleic acids [112]. Efficient release of the polyplex into the cytoplasm is furthermore enhanced by the proton sponge effect of PEI that helps in endosomal escape [141]. However, the formation of aggregates through intracellular protein interaction is an issue due to its non-biodegradable nature within the cells, as a result of which dose-dependent cytotoxicity is a major concern [142]. In order to reduce the cytotoxicity and enhance transfection efficiency, studies have been conducted on PEI nanocarrier modifications using poly (L-lysine) (PEI-PLL), polyurethane (PU-PEI), poly(1,8-octanediocitric acid)-co-polyethylene glycol grafted with PEI (POCG-PEI), and PLGA/cetylated PEI/hyaluronic acid nanoparticles (PCPH nanoparticles) [143,144,145,146]. In a study by Wang et al. on biodegradable poly(1,8-octanedio-citric acid)-co-polyethylene glycol grafted with PEI (POCG-PEI) for nucleic acid delivery, it was found that this POCG-PEI can bind with siRNA and miRNA as a protective shield from enzymatic degradation, along with enhanced cellular uptake and significant reduction in cytotoxicity as compared to that of unmodified PEI [147]. In another study by Son et al., the introduction of a disulfide (-S-S-) linkage in the branched PEI (SSPEI) enhanced the biodegradability of the polymer and thereby achieved better release of the nucleic acid indirectly with the help of endogenous enzymes [148]. Furthermore, Li et al., by coupling poly (ε-caprolactone) (PCL) to PEI via bio-cleavable disulfide linkage, synthesized an amphiphilic cationic graft polymer called PSSP (polyethyleneimine-cystamine-poly(ε-caprolactone), which showed high transfection efficiencies and low cytotoxicity along with intracellular redox potential to trigger miRNA release [149]. Apart from the PEI modifications, studies based on the use of low-molecular-weight PEI showed better biocompatibility and degradability along with a lower charge density, thus leading to a lower degree of membrane damage and lower cytotoxicity compared to that of the higher-molecular-weight PEI [150]. With regard to pain-related studies conducted using PEI, a study by Cheng et al. using an miR-195 mimic encapsulated in jetPEI, a commercial PEI-based nanoparticle, proved that miR-195-treated stroke rats showed antiapoptotic characteristics via inhibiting Sema3A/Cdc42/JNK signaling and inflammation reduction [151].

##### Polyamidoamine (PAMAM) Dendrimer-Based Nano-Systems

Dendrimers are hyperbranched polymers with symmetrical structures having a central core surrounded by three or more branches of repeating monomer units that have terminal functional groups at the ends of the branches for conjugation [152,153]. One of the main advantages of dendrimers is that the size, shape, charge, and solubility can be controlled by manipulating the monomer units [154]. PAMAM dendrimers are a group of dendrimers widely used to facilitate miRNA delivery based on their ability to conjugate ligands for targeted cell specificity [155,156,157]. PAMAM has a radial structure with primary amine functional groups on its surface and a diaminobutane core [158,159]. The combination of the surface amines and interior amide linkages offers better biocompatibility for PAMAM dendrimers over other types. However, cytotoxicity and low transfection efficiency are major limiting factors, which can only be overcome by surface modification and alteration of generation [160,161,162]. Studies based on modifying the dendrimer generation and surface functional groups were conducted by Zeng et al.’s group on neuronal differentiation using human progenitor cells, and Wu et al. synthesized aptamer-modified PEGylated PAMAM [163,164]. PAMAM can be used to co-deliver two different molecules such as antagomir-21 and 5-fluorouracil and can also be coupled with other moieties to enable multifaceted therapy and imaging as in the case of gadolinium-functionalized nanographene oxide (Gd-NGO) with PAMAM [165,166].

#### 4.2.2. Inorganic Nano-Systems

The production of structures having a tunable size, morphology, and composition along with the simplicity in formulation procedures makes synthetic or inorganic nanomaterials potential delivery vehicles for miRNA delivery [167,168]. Nucleic acid incorporation can be achieved via encapsulation, adsorption, or covalent adsorption using particle surface modifications with reactive species [169,170]. One of the most commonly used inorganic nanomaterial is silica or mesoporous silica nanoparticles. Other promising inorganic nano-systems include gold nanoparticles and iron oxide nanoparticles; however, currently, there are no studies yet conducted on gold or iron oxide nanoparticle-based delivery of miRNA for neuropathic pain treatment.

Studies on the chemical and mechanical stability of gold nanoparticles demonstrates that they are a suitable delivery system for miRNA or other nucleic acids [171]. The negatively charged miRNA can be loaded onto the surface of the gold nanoparticles upon functionalization with thiol or amino groups [91,172]. Another study by Sukumar et al. shows that polyfunctional gold–iron oxide nanoparticles or polyGION nanoparticles could be surface functionalized with chitosan–cyclodextrin (CD-CS) hybrid polymers, which increased the surface potential from −15 mV to +39 mV due to the excess free positive charges of the amine groups of chitosan, thereby giving it a loading efficiency of 80% [170]. However, gold nanoparticles of 5 nm may disrupt the fibroblast structure after 72 h of exposure, thereby limiting the clinical application due to the potential cytotoxicity [173].

Iron oxide nanoparticles are largely used owing to the magnetic properties of its core which enables it to be used for magnetic resonance imaging (MRI), in addition to its theranostic capabilities of delivering nucleic acids [165]. SPIONs or super paramagnetic iron oxide nanoparticles are often used as contract agents in gene therapy. SPIONs are coated with polymers to stabilize and enhance the biocompatibility, and with lipids to evade RES clearance and the immune response [174,175]. Even though direct studies on iron oxide nanoparticle-based delivery of miRNA for chronic neuropathic pain are scarce, these listed studies highlight the conceptual frameworks indicating the potential applications and pre-clinical approaches that could be extended to chronic pain. The properties of iron oxide nanoparticles such as magnetic targeting and theranostic potential along with high cargo capacity and stability can be utilized for the delivery of miRNA such as miR-146a which can downregulate NF-κB-associated signaling (IRAK1, TRAF6) and reduce pro-inflammatory cytokine release [176]. In the case of miR-124 delivery, they can possibly convert activated M1 macrophages towards the M2 phenotype and alleviate DRG neuroinflammation and attenuate hyperalgesia and allodynia [177].

##### Silica Nanoparticles

Silica nanoparticles, especially mesoporous silica nanoparticles (MSNs) having a diameter of 100–200 nm are widely used in miRNA delivery due to their simplicity in functionalization, high porous architecture, biocompatibility, and the biodegradable properties [171,178]. Mesoporous silica nanoparticles have the ability to simultaneously deliver and also to have a controlled release of miRNAs and small molecules at the target sites [179]. Large surface areas, high loading capacities, chemically modifiable surfaces along with low toxicity make it a potential delivery system [180]. Pore size plays a major role in both the release rate as well as loading. MSNs with a smaller pore size can provide a tunable release rate for the oligonucleotide, whereas a larger pore size enables it to have a faster release rate and a higher loading capacity [181]. Hosseinpour et al. studied the delivery of miRNA-26a to macrophages to promote M2 polarization via mesoporous core-cone silica nanoparticles [182]. In the study, murine-derived macrophage cell lines and preosteoblastic murine-derived cell lines from a mouse were treated with miRNA-26a-loaded mesoporous. In comparison to polymeric nanoparticles and liposomes, MSNs are more resistant and have the ability of sustained release over time, making it an optimum vehicle for solving problems related to sustained drug release and targeted delivery. Recent studies by Yitian Lu et al. demonstrated that a surface-aminated MSN-mediated miR-26a-5p delivery system exhibited excellent anti-inflammatory effects, effectively reduced microglial activation, and proved to be an effective analgesic for inflammatory- and chemotherapy-induced peripheral neuropathic pain [100].

#### 4.2.3. Lipidic Nano-Systems

Lipid-based nano-delivery systems consisting of a polar head group, hydrophilic tail region, and domain linker along with other lipid components such as cholesterol, phospholipids, and PEG have stable nanostructures in physiological fluids and have the ability to fuse with negatively charged endosomal membranes, thus making it an effective nucleic acid delivery system [183]. Even though several studies have been conducted for miRNA delivery using lipid-based nanoparticles such as liposomes, solid lipid nanoparticles (SLNs) and hybrid lipid–polymer nanoparticles, they are the most widely used methodologies for the in vivo delivery of small interfering RNAs or siRNAs [184,185]. The simplicity and safety features of lipid-based nanocarriers make them one of the most widely used delivery platforms for miRNA [186,187,188,189]. Lipid-based nanoparticles can protect the oligonucleotides from enzyme degradation, promote cellular uptake, and enhance the circulation half-life [190]. In addition to that, SLN and hybrid lipid–polymer nanoparticles have not yet been used in the delivery of miRNA in neuropathic pain treatment.

There are a very few studies based on SLNs used to co-deliver an oligonucleotide and a drug that offer enhanced therapeutic activity due to the nano-structural properties. In one study, anti-miR-21 and pemetrexed were encapsulated into SLNS which helped in higher cellular uptake when compared to that of the free drug [191]. Similarly, in another study, the synergistic effects of miR-34a and paclitaxel were exploited in an SLN co-delivery system [192]. Furthermore, Shi et al. synthesized another cationic SLN composed of dimethyl-dioctadecyl ammonium bromide (DDAB), glyceryl monostearate, polyoxythylene 50 stearate, cholesterol, and soy phosphatidylcholine via film ultrasonic techniques and loaded with miRNA via incubation [193]. Although these studies are primarily on cancer research, SLNs prove to be promising vectors for miRNA delivery, which in turn can be potentially used for chronic pain treatment too.

Lipopolyplexes or hybrid lipid–polymer nanoparticles comprise nucleic acids encapsulated or complexed with a biodegradable polymer that offer protection in a lipid bilayer which allows easy targeting and stealth moiety for a longer half-life in blood [194]. A study by Huang et al. based on lipopolyplexes consisting of PEI and dioleoylphosphatidyl ethanolamine (DOPE)/linoleic acid/1,2-dimyristoyl-rac-glycero-3-methoxypolyethylene glycol-2000 (DMG-PEG) on which transferrin molecules were post inserted for miR-29b delivery showed better efficiency, while the same formulation for miR-1 transferrin-mediated delivery showed 3-fold higher efficiency comparatively [195,196]. Only a few studies have been performed on pain models using lipopolyplexes, but nevertheless, it is a promising carrier. A recent study based on cationic lipid 1,2-dioleoyl-3-trimethylammonium propane (DOTAP) and DOPE together with miR-155 formed a lipoplex that was used in the treatment of intervertebral disc (IVD) degeneration which corresponds to 40% of cases of lower back pain [197].

##### Cationic Lipid Nanoparticles (LNPs)

A typical cationic lipid-based nanoparticle formulation consists of cationic lipids and helper lipids such as neutral lipids and PEG-lipids. Cationic liposomes or lipid nanoparticles commonly used for miRNA delivery are 1,2-di-O-octadecenyl-3-trimethylammonium propane (DOTMA), DOTAP, DDAB, and 1,2-dioleyloxy-3-dimethylaminopropane (DODMA) [198,199,200,201]. LNPs also possess additional units called helper lipids such as cholesterol and DOPE to increase the stability and reduce the toxicity and PEG to reduce the nanoparticle aggregation and increase the circulation time [202,203,204]. Lipid nanoparticles can also be developed from ionizable cationic lipids having primary, secondary, or tertiary amines as the headgroup domain with pKa values below 7. Based on a study conducted by Yung et al., lipid nanoparticles having tertiary and quaternary amine groups were prepared for therapeutic delivery of miR-21 antagomiR [205]. Even though here, this study relates to cancer treatment, miR-21 antagomiR is commonly used in rat models and blocks miR-21 and alleviates neuropathic pain via the reduction in allodynia and hyperalgesia and lowers neuroinflammatory markers.

**Figure 7:**
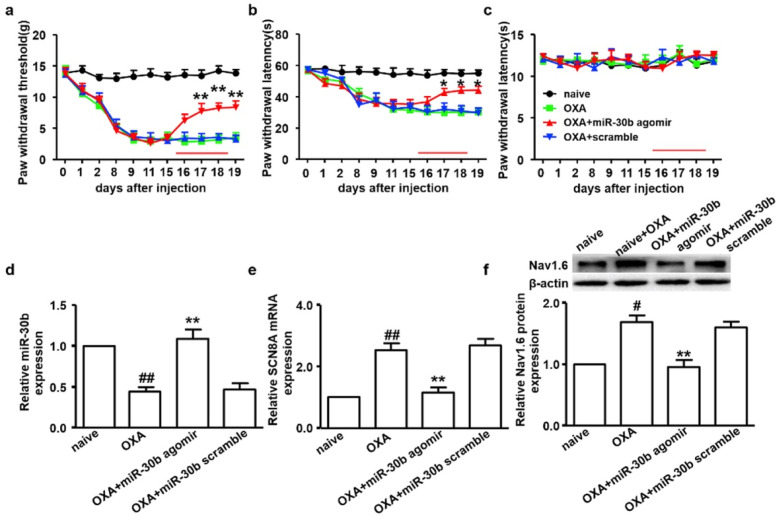
Intrathecal administration of miR-30b agomir inhibits the expression of Na_v_ 1.6 in DRG neurons and attenuates neuropathy. Figures indicate paw-withdrawal thresholds in response to (**a**) mechanical stimuli, (**b**) cold stimuli, and (**c**) thermal stimuli after over-expression of miR-30b. * *p* < 0.05 and ** *p* < 0.01 vs. oxaliplatin group. (**d**) The relative expression of miR-30b was determined by RT-qPCR in oxaliplatin-injected rats after intrathecal administration of miR-30b agomir. ** *p* < 0.01 vs. oxaliplatin group, ## *p* < 0.01 vs. naïve group. Na_v_ 1.6 (**e**) mRNA and (**f**) protein expression were decreased in oxaliplatin-injected rats after intrathecal administration of miR-30b agomir. ** *p* < 0.01 vs. oxaliplatin group. # *p* < 0.05. (reprinted from [67] with permission from Elsevier).

Lipofectamine, a common transfection reagent consisting of lipid subunits and forms liposomes in aqueous environment, is often used to entrap nucleic acid payloads such as mRNA, siRNA, miRNA, and plasmid DNA. It is a 3:1 mixture of DOSPA (2,3-dioleoyloxy-N-[2(sperminecarboxamido)ethyl]-N,N-dimethyl-1-propaniminium trifluoroacetate) and DOPE [206]. Li et al. used lipofectamine-based miR-30b-5p transfection to attenuate oxaliplatin-induced peripheral neuropathic pain via Na_v_ 1.6 VGSCs in male Sprague-Dawley rats (Figure 7) [67]. In another study, the upregulation of miR-96 using lipofectamine transfection was found to alleviate neuropathic pain in CCI rat models by inhibiting Na_v_ 1.3 channels in the DRG [32]. Furthermore, studies also highlight the role of potassium voltage-gated channels and miRNA in alleviating neuropathic pain. Zhang et al. studied the influence of lipofectamine-transfected miR-137 agomir/antagomir and K_v_1.2 channels on CCI-induced rat models, which demonstrated that the downregulation of miR-137, upregulation of Kcna2, and gene encoding in the K_v_1.2 channel alleviate neuropathic pain [34]. Intrathecal injection of lipofectamine/miR-140 on CCI rat models in a study conducted by Li et al. demonstrated the attenuation of neuropathic pain by targeting Sphingosine-1-phosphate receptor 1 (S1PR1) [35]. Studies by Guangyao et al. proved the involvement of the Na_v_ 1.3 channel encoded by the sodium voltage-gated channel alpha subunit 3 (SCN3A) gene, an isoform of tetrodotoxin-sensitive (TTX-S) in CCI rat models and the fact that negative regulation of it using lipofectamine-transfected miR-384-5p ameliorates neuropathic pain both in vivo and in vitro [45]. The regulation of the Na_v_ 1.7 channel encoded by the SCN9A gene in a spared nerve injury (SNI) rat model by using Invivofectamine™ 3.0 (Invitrogen, Carlsbad, CA, USA) transfected miR-182 in neuropathic pain treatment was conducted by Cai et al. [68].

#### 4.2.4. Extracellular Vesicles-Based Nano-Systems

**Figure 8:**
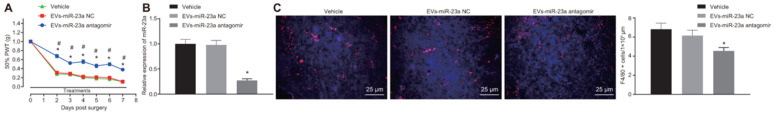
Delivery of miR-23a antagomir reduced neuropathic hypersensitivity and recruitment of M1 macrophages in vivo (**A**) mechanical hypersensitivity; * *p* < 0.05 vs. vehicle mice; # *p* < 0.05 vs. oligomer-treated mice. (**B**) RT-qPCR analysis of miRNA expression in DRG. (**C**) Immunostaining of macrophages (red) and nuclei (blue) in SNI DRG; * *p* < 0.05 vs. mice treated with EVs-miR-23a-NC or vehicle (reprinted from [29]).

Extracellular vesicles (EVs) are nanometer-scale carriers composed of phospholipid bilayer-based vesicles that are secreted from all cell types and commonly found in biological fluids such as saliva, blood, breast milk, cerebrospinal fluids, and malignant ascites [207,208]. Owing to the unique biological characteristics along with their role in cell-to-cell communication, EVs have attracted strong interest in therapeutics. Based on their biogenesis, EVs can be classified as exosomes, microvesicles, and apoptotic bodies [209]. The size of an exosome ranges from 40 to 120 nm while that of microvesicles are in the range of 50 to 1000 nm [210,211]. EVs function as a carrier vehicle for infectious particles, oncogenes, and biomolecules such as lipids, proteins, DNA transcription factors, mRNAs, and small or large non-coding RNAs, especially miRNAs between various cell types [212,213]. Over conventional nanocarriers for drugs and therapeutic agents, EVs have advantages such as good biocompatibility, low immunogenicity, capacity for gene delivery, and natural blood–brain barrier penetration [214]. Post peripheral nerve injury, neuro–immune interactions occur in the DRG resulting in the release of EVs containing miRNAs, which become engulfed by macrophages leading to alterations affecting the pain mechanisms [215]. Studies reveal that the inhibition of microRNA23a or miR23a has potential therapeutic intervention for nociceptive hypersensitivity induced as a result of peripheral nerve injury such as an SNI which contributes to enhanced M1 polarization along with the secretion of inflammatory factors [216,217]. Zhang et al. further studied the delivery of extracellular vesicle-encapsulated miR-23a from DRG neurons and its subsequent contribution to the inflammatory response and M1 macrophage polarization in SNI-based neuropathic pain models (Figure 8) [29]. In another investigation by Zhang et al., it was found that exosome-encapsulated miR-181c-5p could attenuate neuropathic pain after sciatic nerve chronic constriction injury [101]. Another study based on the activation of microglia via miR-16-5p knockdown in DRG-derived exosomes by Xing et al. demonstrated the alleviation of neuropathic pain [102].

#### 4.2.5. Hydrogel-Based Systems

Hydrogels are a 3-dimensional network with polymer crosslinking that has the ability to absorb water and also retain it [218]. Hydrogels provide a natural aqueous environment for biomolecules by encapsulating them to function in biological systems and provide a diffusion barrier that allows the passage of molecules of a certain threshold only, thus protecting the biological agents from immune rejection or degradation [219]. Due to its ideal injectable characteristics along with controllable degradability, good mechanical properties, and negligible cytotoxicity, hydrogels are often considered as one of the best candidates for drug delivery [220]. In addition to that, the tunability of the stiffness of hydrogels from 0.5 kPa to 5 MPa allows their physical properties to match those of various soft tissues in the human body [221,222,223]. Hydrogels can be classified into three main categories depending on the requirements of the delivery route, such as macroscopic hydrogels, microgels, and nanogels. Microgels and nanogels are smaller hydrogel particles often used as a minimal invasive delivery route owing to its injectable properties, large surface area for bioconjugation, enhanced tissue penetration, and facile natural clearance [224]. Nanohydrogels or nanogels possess a very high surface to volume ratio thereby facilitating cross cellular membrane transfer and efficient cell uptake [225]. Natural polymeric-based nanohydrogels offer advantages such as high biodistribution, non-cytotoxic nature, biodegradability, and high clearance rates. However, they often suffer from uncontrolled structures and unpredictable drug release behavior. These limitations can be addressed by employing synthetic polymeric systems involving materials such as polyamides, PEG, polypeptides, and polyesters [226,227]. Synthetic polymeric-based nanohydrogels demonstrate high stability, controlled architectures, and tunable drug release properties, and are generally designed to exhibit low immunogenicity [228,229]. Nevertheless, minor immune responses can still arise depending on the polymer type, surface modifications, and degradation products. Therefore, by incorporating bioactive probes or by developing hybrid hydrogels that combine both natural and synthetic polymers, it is possible to optimize biocompatibility, minimize immunogenicity, and achieve controlled therapeutic delivery [218].

Hydrogels are often used in gene therapy due to their tunable physical characteristics and controlled release of the nucleic acid drugs. Other advantages of using hydrogels for gene delivery include fewer off-target effects, the maintenance of bioactivity, the possibility of local administration, and the avoidance of multiple administration and on-demand or pulsatile as well as prolonged release of the drugs [230]. Nucleic acids such miRNA can be either loaded directly or by encapsulating it in a nanocarrier system. Nanocarrier loading provides better controllability and specific targeting of cells along with improved bioactivity comparatively. MiRNA-based hydrogels have been studied for cancer therapy, immunomodulation, bone regeneration, cardiovascular disease, spinal cord injury, intervertebral disk degeneration, and more [231,232,233,234,235,236]. Studies by Li et al. on the hydrogel-based controlled delivery of miR-21-5p using mesoporous silica nanoparticles shows an anti-inflammatory response in porcine models by inhibiting the macrophage polarization to M1 [237]. In another study, Wang et al., based on work on curcumin and cholesterol-modified antagomir-21 delivery for intervertebral disc degeneration treatment using an injectable inflammation-responsive gelatin-based hydrogel, demonstrated anti-inflammatory activity by inducing the M2 phenotype polarization of macrophage cells in vitro [238].

## 5. Conclusions and Future Perspectives

In the recent years, significant scientific contributions have been made in understanding the crucial role of miRNAs in the pathogenesis of chronic neuropathic pain conditions. Studies on miRNA have increased exponentially ever since the demonstration of its use as a therapeutic agent and a biomarker. The regulation of gene expression related to neuroinflammation, neuronal excitability, and synaptic plasticity is only one among the pivotal roles that these short non-coding RNAs play. Nerve injury-induced miRNA dysregulation and subsequent macrophage polarization could be regulated to induce chronic neuropathic pain treatment. Despite these insights, the clinical translation of miRNA-based therapies faces challenges with regard to safe, effective, and successful delivery systems for miRNAs to target tissues.

Drug delivery systems for miRNA-based cancer treatment are relatively more common compared to that for neuropathic pain treatment. Even though recent progress in the formulation of various delivery systems is promising, the formulation of an ideal vector is yet miles away. Limitations and drawbacks from the current data can be used in formulating a better nanocarrier for nucleic acid delivery. Current limitations include miRNA instability in vivo, off-target effects, immune reactions, and lack of DRG-specific targeting. To address these, future research should emphasize stimuli-responsive and multifunctional carriers (e.g., pH- or enzyme-sensitive nanoparticles) and targeted delivery (e.g., with DRG-homing ligands). The co-delivery of miRNA combinations or miRNA plus drugs may enhance the efficacy while minimizing doses. Innovative strategies like hybrid nanocarriers (combining polymer and lipid components), surface engineering, and triggered release systems could help overcome the stability and specificity challenges. In conclusion, while miRNA-based therapies outline and represent a promising frontier in chronic neuropathic pain treatment, the development of efficient and safe systems remains a crucial hurdle. The clinical application and translation face key challenges, including immune response concerns and regulatory approvals. Notably, miR-146a-loaded PLGA nanoparticles were shown to reduce neuroinflammation in rodent pain models; however, translating this into human trials requires addressing nanoparticle stability and precise dosing. Future research should focus on standardized protocols and clinical validation, with miR-146a-loaded PLGA nanoparticles. Currently, no miRNA-based pain therapies have entered Phase III clinical trials. Ongoing research into innovative delivery mechanisms involving advanced nanoparticle platforms is very much essential in harnessing the full therapeutic potential of miRNAs, in order to translate such findings into clinical practice.

## Figures and Tables

**Table 1:** Neuropathic pain-associated microRNA regulation at DRG.

miRNA	Regulation	Injury Model	Reference
miR-7a	Down	Spinal nerve ligation	Sakai et al. [26]
miR-17-92 cluster	Up	Spinal nerve ligation	Sakai et al. [27]
miR-21	Up	Spinal nerve ligation	Zhang et al. [28]
miR-23a	Up	Spared nerve injury	Zhang et al. [29]
miR-30b	Down	Spared nerve injury	Shao et al. [30]
miR-34a	Down	Chronic constriction injury	Brandenburger et al. [31]
miR-96	Down	Chronic constriction injury	Chen et al. [32]
miR-124	Down	Spared nerve injury	Willemen et al. [33]
miR-137a	Up	Chronic constriction injury	Zhang et al. [34]
miR-140	Down	Chronic constriction injury	Li et al. [35]
miR-141	Down	Chronic constriction injury	Zhang et al. [36]
miR-142-3p	Down	Spinal nerve ligation	Zhang et al. [37]
miR-144	Down	Chronic constriction injury	Zhang et al. [38]
miR-146a-5p	Up	Chronic constriction injury	Wang et al. [39]
miR-150	Down	Chronic constriction injury	Cai et al. [40]
miR-182	Down	Chronic constriction injury	Jia et al. [41]
miR-183	Down	Chronic constriction injury	Shi et al. [42]
miR-216	Down	Chronic constriction injury	Wang and Li [43]
miR-381	Down	Chronic constriction injury	Xia et al. [44]
miR-384	Down	Chronic constriction injury	Ye et al. [45]
miR-494-3p	Down	Spared nerve injury	Zhang et al. [46]
miR-590-3p	Down	Diabetic neuropathic pain	Wu et al. [47]

**Table 2:** Key limitations or challenges of delivering naked miRNA vs. encapsulated miRNA delivery using carriers.

Characteristics	Naked miRNA	miRNA Encapsulation
Cell uptake	Cell uptake is hindered due to negatively charged miRNA 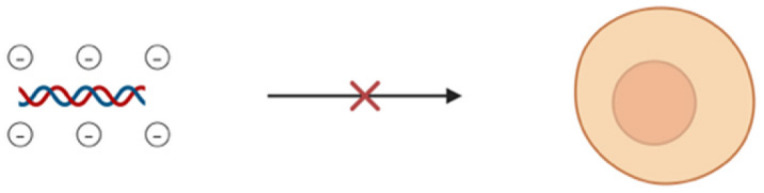	Negative charges are shielded when encapsulated by cationic delivery systems and thereby, there is no hinderance in cell uptake 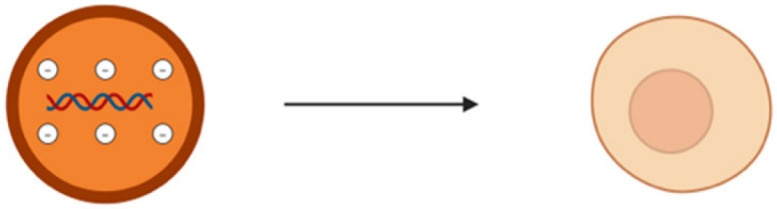
Target	It may potentially harm the healthy cells or go off-target 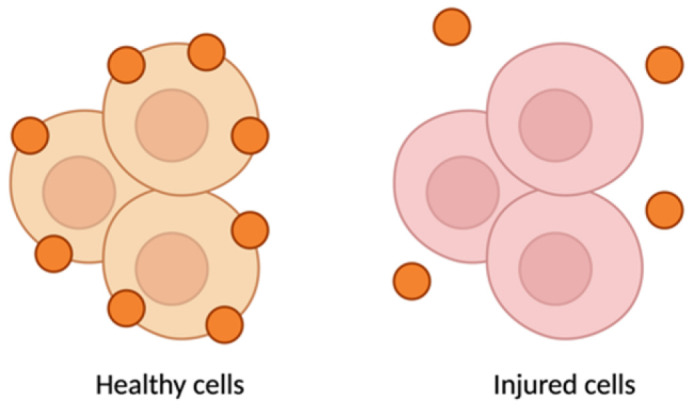	It is target specific with modifications as per requirements 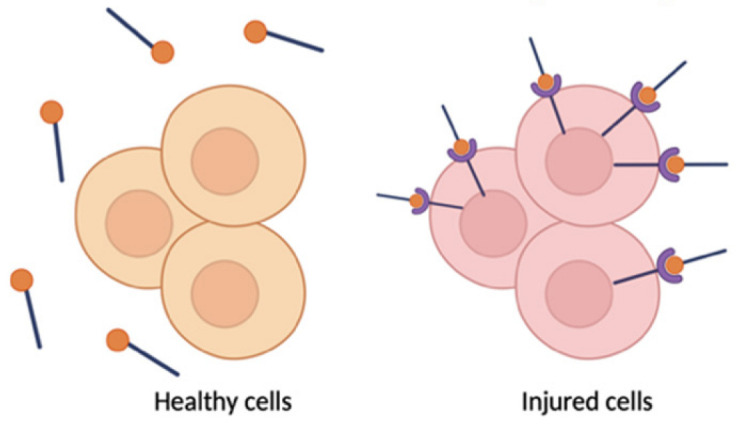
Release	Bolus administration results in the excessive activation of genes 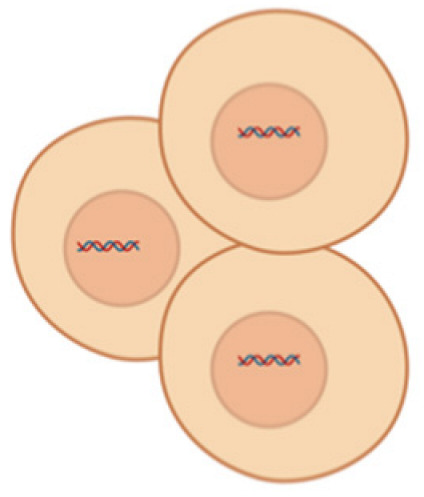	Sustained delivery/release of miRNA instead of bolus 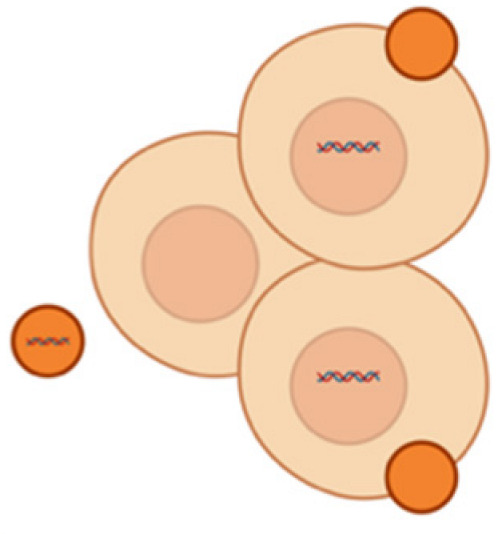
Half-life	Short half-life due to nuclease 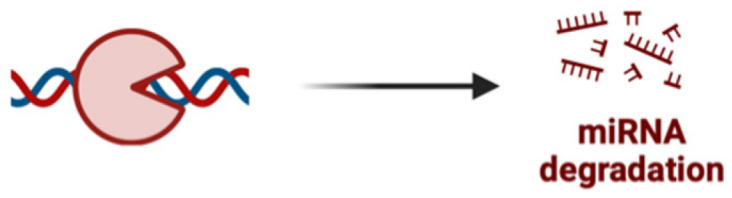	Protected against nuclease degradation 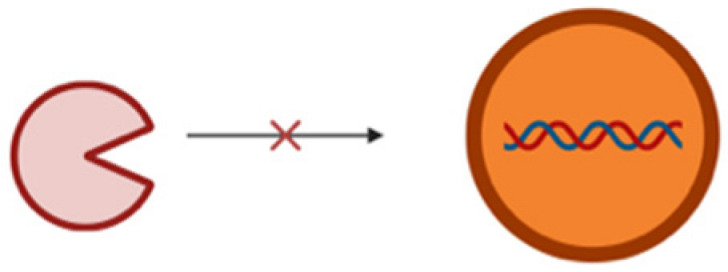
Immune response	RES clearance or immune cell activation 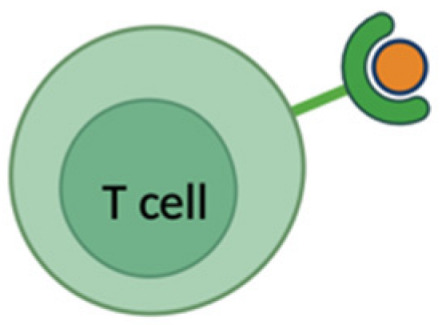	Stealth coating prevents immune response generation 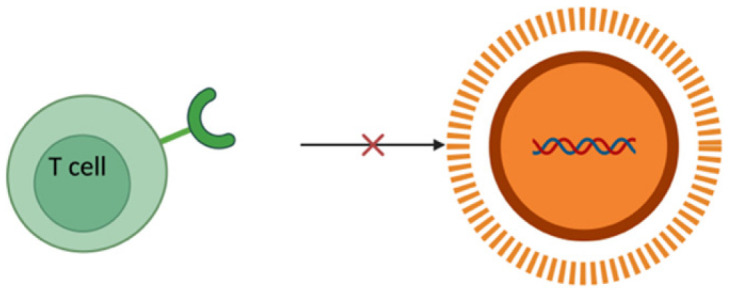
Legend: 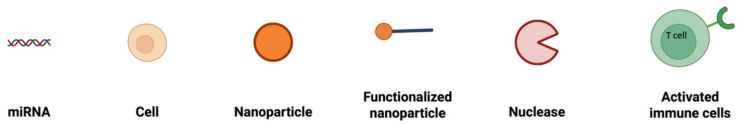		

**Table 3:** Current list of miRNA delivery systems for chronic neuropathic pain treatment at DRG.

Delivery System	miRNA	Injury Model	Cell Lines/In Vivo	Reference
Lentivirus	miR-141	CCI	Rat DRG neurons	[36]
	miR-142-3p	SNL	ICR Mice	[37]
	miR-144	CCI	DRG/glial cells from CCI mice	[38]
	miR-145	CCI	Rat adrenal pheochromocytoma cell line PC12	[96]
	miR-150	CCI	Female Sprague-Dawley rats/Rat microglial cells/HEK-293T cells	[97]
	miR-183-5p	CCI	Male Sprague-Dawley rats	[42]
	miR-216a-5p	CCI	Male Sprague-Dawley rats	[43]
Adeno-associated virus	miR-7a	SNL	Male Sprague-Dawley rats/Rat DRG neurons	[98]
	miR-7a	SNL	Male Sprague-Dawley rats/Rat DRG neurons	[26]
	miR-17-92	SNL	Male Sprague-Dawley rats	[27]
Herpes Simplex virus	QHmiNa_v_	DIPDN	Rat embryo DRG neurons/Male Sprague-Dawley rats	[99]
Silica nanoparticles	miR-26a-5p	SNI	BV2/DRG cell lines	[100]
Cationic lipid nanoparticles	miR-30b	OINP	Male Sprague-Dawley rats	[67]
	miR-96	CCI	Rat DRG neurons	[32]
	miR-137	CCI	Male Sprague-Dawley rats	[34]
	miR-140	CCI	HEK293	[35]
	miR-384-5p	CCI	Male Sprague-Dawley rats/DRG neurons/HEK 293T cells	[45]
	miR-182	SNI	Male Sprague-Dawley rats	[68]
Extracellular vesicles	miR-23a	SNI	Mice DRG neurons	[29]
	miR-181c-5p	CCI	Male Sprague-Dawley rats	[101]
	miR-16-5p	SNL	Adult and naïve male C57BL/6J mice	[102]

## List of Abbreviations

AAV	Adeno-associated virus
Ca_v_	Calcium channels
CD	Cyclodextrin
CSF	Cerebrospinal fluid
DCM	Dichloromethane
DMG-PEG	1,2-dimyristoyl-rac-glycero-3-methoxypolyethylene glycol-2000
DODMA	1,2-dioleyloxy-3-dimethylaminopropane
DOPE	Dioleoylphosphatidyl ethanolamine
DOSPA	2,3-dioleoyloxy-N-[2(sperminecarboxamido)ethyl]-N,N-dimethyl-1-propaniminium trifluoroacetate
DOTAP	1,2-dioleoyl-3-trimethylammonium propane
DOTMA	1,2-di-O-octadecenyl-3-trimethylammonium propane
DRG	Dorsal root ganglia
EMA	European Medicine Agency
EVs	Extracellular vesicles
FDA	Food and Drug Administration
Gd-NGO	Gadolinium-functionalized nanographene oxide
GRAS	Generally recognized as safe
HMGB1	High-mobility group box 1
HSV	Herpes simplex virus
IASP	International Association for the Study of Pain
IVD	Intervertebral disc
K_v_	Potassium channels
miRNA	Micro-RNA
MRI	Magnetic resonance imaging
mRNA	Messenger RNA
MSNs	Mesoporous silica nanoparticles
mTOR	Mechanistic/Mammalian target of rapamycin
N/P ratio	Amine to phosphate ratio
Na_v_	Sodium channels
ncRNA	Non-coding RNA
NF-κB	Nuclear factor kappa B
PAMAM	Poly(amidoamine)
PCL	Poly (ε-caprolactone)
PCPH	PLGA/cetylated PEI/hyaluronic acid
pDMAEMA	Poly (2-dimethylamino) ethyl methacrylate
PDN	Painful diabetic neuropathy
PEG	Poly (ethylene glycol)
PEI	Polyethyleneimine
PGA	Poly-glycolic acid
POCG	Poly(1,8-octanediocitric acid)-co-polyethylene glycol
polyGION	Polyfunctional gold-iron oxide nanoparticles
PSN	Primary sensory neurons
PSSP	Polyethyleneimine-cystamine-poly(ε-caprolactone)
PU	Polyurethane
PVA	Poly (vinyl alcohol)
PWL	Paw withdrawal latency
PWT	Paw withdrawal threshold
RES	Reticuloendothelial system
RISC	RNA-induced silencing complex
-S-S-	Disulfide
S1PR1	Sphingosine-1-phosphate receptor 1
SCI	Spinal cord injury
SCN3A	Sodium voltage-gated channel alpha subunit 3
siRNA	Small interfering RNA
SLNs	Solid lipid nanoparticles
SNI	Spared nerve injury
SNL	Spared nerve ligation
SPIONs	Super paramagnetic iron oxide nanoparticles
STAT3	Signal transducer and activator of transcription 3
STZ	Streptozotocin
TA	Tragacanthic acid
TPP	Tripolyphosphate
TRBP	Trans-activation response RNA-binding protein
TREK1	TWIK-related potassium channel 1
TTX-S	Tetrodotoxin-sensitive
VGSC	Voltage-gated sodium channels
W/O/W	Water in oil in water
XIST	X-inactive specific transcript
